# Use of bevacizumab as a single agent or in adjunct with traditional chemotherapy regimens in children with unresectable or progressive low‐grade glioma

**DOI:** 10.1002/cam4.1799

**Published:** 2018-12-19

**Authors:** Nataliya Zhukova, Revathi Rajagopal, Adrienne Lam, Lee Coleman, Peter Shipman, Thomas Walwyn, Molly Williams, Michael Sullivan, Martin Campbell, Kanika Bhatia, Nicholas G. Gottardo, Jordan R. Hansford

**Affiliations:** ^1^ Children’s Cancer Centre The Royal Children’s Hospital Melbourne Victoria Australia; ^2^ Department of Pediatric and Adolescent Clinical Hematology and Oncology Perth Children’s Hospital Perth West Australia Australia; ^3^ Department of Pediatrics University Malaya Medical Center Kuala Lumpur Malaysia; ^4^ Department of Radiology The Royal Children’s Hospital Melbourne Victoria Australia; ^5^ Department of Pediatrics University of Melbourne Melbourne Victoria Australia; ^6^ Murdoch Children’s Research Institute Melbourne Victoria Australia; ^7^ Department of Radiology Perth Children’s Hospital Perth West Australia Australia

**Keywords:** bevacizumab, brain tumor, cancer, humanized monoclonal antibody, pediatric low‐grade glioma, vascular endothelial growth factor

## Abstract

In pediatric low‐grade gliomas not amenable to complete resection, various chemotherapy regimens are the mainstream of treatment. An excellent overall survival of these patients makes justification of the intensification of chemotherapy difficult and calls for the development of new strategies. Bevacizumab, a humanized monoclonal antibody directed against Vascular endothelial growth factor (VEGF), has been successfully used in combination with irinotecan in a number of adult and pediatric studies and reports. Fifteen patients at median age of 7 years old (range 3 months to 15 years) were treated with bevacizumab in combination with conventional low‐toxicity chemotherapy. The majority had chiasmatic/hypothalamic and midline tumors, seven had confirmed BRAF pathway alterations including neurofibromatosis type 1 (2). Fourteen patients had more than one progression and three had radiotherapy. No deaths were documented, PFS at 11 and 15 months was 71.5% ± 13.9% and 44.7% ± 17.6% respectively. At the end of follow‐up 40% of patients has radiologically stable disease, three patients progressed shortly after completion of bevacizumab and two showed mixed response with progression of cystic component. Rapid visual improvement was seen in 6/8 patients, resolution of endocrine symptoms in 2/4 and motor function improvement in 4/6. No relation between histology or BRAF status and treatment response was observed. Treatment‐limiting toxicities included grade 4 proteinuria (2) and hypertension (2) managed with cessation (1) and pausing of therapy plus antihypertensives (1). In conclusion, bevacizumab is well tolerated and appears most effective for rapid tumor control to preserve vision and improve morbidity.

## 1 INTRODUCTION

### 1.1 Pediatric low‐grade gliomas

Central nervous system (CNS) tumors are the most prevalent malignancies in children and young adults after leukemias^1^ and the 4th most common cause of death in children and young adults,^2^ with gliomas of all grades accounting for 50% of all CNS malignancies.^2^, ^3^ Pediatric low‐grade gliomas (PLGG), defined by World Health Organization (WHO) based on their histological features as grade I and II tumours, comprise 2/3 of gliomas in the 0‐19 age group.^2^, ^3^, ^4^, ^5^, ^6^ Pilocytic astrocytomas (PA) are the most common form, but other tumor types include pilomyxoid astrocytoma, low‐grade oligoastrocytoma, low‐grade oligodendroglioma, mixed low‐grade glioma, and ganglioglioma.^6^, ^7^ Although surgery is typically curative for lesions amenable to complete resection, PLGGs are challenging tumors to treat when they are unable to be resected. They often arise deep in the brain or in close proximity to vital structures, making complete surgical resection difficult.^8^ Their behavior is often indolent, with multiple progressions and varying degrees of chemosensitivity.^9^, ^10^

Historically, external beam radiation was effective therapy for unresectable or incompletely resected tumors.^11^ However, due to the tendency of these lesions to be large and to typically affect young children, the potential for radiation‐induced brain injury, with the associated serious cognitive, developmental, and endocrine sequelae in the developing brain, is of major importance. As a result, alternative therapeutic strategies are currently used as first‐line treatment, reserving radiation for tumors that have progressed despite the use of multiple lines of chemotherapy.^7^, ^12^ A strategy combining close observation with systemic chemotherapy if necessary has become the standard of care.^13^

Tumor progression, threat to vision and hypothalamic‐pituitary dysfunction in the case of an optic pathway glioma, tumor location, residual disease, age of the patient, and association with neurofibromatosis type 1 (NF1) can influence the decision about particular chemotherapeutic regimens.^14^ Currently, numerous protocols are available to halt progression or delay the need for potentially damaging radiation treatment. These chemotherapy regimens are generally nonspecific and associated with numerous short and long‐term side effect profiles. A number of protocols have reported varying, although not significantly different, outcomes of 34%‐51% for PFS and 86%‐97% OS.^15^, ^16^, ^17^, ^18^ However, across all chemotherapy protocols, the 5‐year PFS for chemotherapy treated children is significantly inferior to the 5‐year PFS for irradiated children, which is 69%‐74%.^11^, ^19^, ^20^, ^21^ The usually chronic nature of PLGGs and the excellent overall survival rates of these patients^7^ make justification of cytotoxic chemotherapy and its associated side effects, such as ototoxicity, peripheral neuropathy, infections and risk of secondary malignancy, difficult and undesirable, and has led to the search for regimens that are better tolerated and that can achieve durable tumor control.

### 1.2 Angiogenesis in pediatric low‐grade glioma

Vascular endothelial growth factor (VEGF) plays a vital role in physiological angiogenesis during embryogenesis and growth.^22^ Increased expression levels of VEGF coinciding with vascular proliferation have been reported in normal rat ovary during formation of corpus luteum^23^ and in developing murine brain tissue^24^ and indicate its important role in endothelial cell proliferation and development of normal microvasculature. There is also ample evidence that VEGF plays a key role in pathological angiogenesis and increased vascular permeability allowing for tumor expansion.^22^, ^25^, ^26^, ^27^ The binding of VEGF to its receptors initiates the signaling pathway that results in new blood vessel formation (neovascularization). Newly formed tumor blood vessels are highly dependent on VEGF for continued viability. Increased levels of VEGF mRNA are found in various tumor tissue, including highly vascular glioblastoma multiforme (GBM).^22^ Importance of VEGF‐mediated neovascularisation in the glial tumor transformation and progression was demonstrated by Jensen and colleagues and later confirmed by Fischer et.al.^26^, ^28^ The critical role of VEGF in angiogenesis has also been made evident in a wide range of other adult and pediatric malignancies.^29^, ^30^, ^31^, ^32^, ^33^ In murine models, anti‐VEGF antibodies have been shown to inhibit tumor growth accompanied by marked reduction in microvasculature formation, but not expansion of tumor cells.^22^, ^34^, ^35^ Pediatric low‐grade gliomas have been shown to express high levels of VEGF, which directly correlate with microvessel density and higher tumor progression rates.^36^

### 1.3 Bevacizumab

Bevacizumab (Roche) is a humanized monoclonal antibody directed against VEGF. It has been used in a number of antiangiogenic‐based regimens. Phase I dose escalation studies established a maximum tolerated dose of 10 mg/kg/dose biweekly.^37^ Earlier studies have demonstrated bevacizumab to have activity in adults with high‐grade gliomas.^38^, ^39^, ^40^, ^41^ More recent reports of small patient series from several institutions have shown that the combination of bevacizumab with irinotecan is effective in children with multiply relapsed low‐grade glioma 42, 43, 44, 45, 46 (Table 1). In addition, bevacizumab has been found effective in patients with optic/chiasmatic gliomas with progressive visual acuity (VA)/visual field (VF) loss despite conventional chemotherapy and radiation.^47^ Bevacizumab‐related toxicities were mild and reversible with the cessation of treatment. These included grade 1‐2 hypertension, fatigue, epistaxis, lymphopenia and grade 1‐4 proteinuria, but no major bleeding, thrombotic complication or wound healing problems were reported in the pediatric population.^42^, ^43^, ^44^, ^45^, ^46^ We now report on the efficacy of bevacizumab in our study population with refractory or progressive PLGG.

**Table 1:** Bevacizumab‐based therapy response rate, progression‐free survival and therapy limiting toxicities in refractory/progressive pediatric low‐grade glioma

Authors	Number of patients	Age at BBT	CR+PR (%)	MR (%)	SD (%)	PFS	Toxicity resulting in discontinuation of bevacizumab
Current study	15	3 mo to 18 y	66.7[Link]	0	0	44.7% ± 17.6% at 15 mo	Grade 4 proteinuria +hypertension (2)
			6.7[Link]	20[Link]	40[Link]		
Kalra et al (2015)	16	1.8‐15.3 y	44[Link]	NA	50[Link]	NA	Grade 2 proteinuria (1)
			19[Link]	0	69[Link]		
Gururangan et al (2014)	35	0.7‐17.6 y	5.7[Link]	NA	17.7[Link]	47.8% ± 9.3% at 2 y	Hypertension +proteinuria (1), proteinuria (3), Fatigue (1), grade 2 epistaxis (2), grade 1 CNS hemorrhage (2), CNS ischemia (1), hip pain (1), knee metaphyseal sclerotic bands (1)
Avery et al (2014)	4	6‐13 y	100[Link]	0	0	NA	Proteinuria (1), hypertension (1), proteinuria +hypertension (1)
Hwang et al (2013)	14	1‐13 y 5 mo	43[Link]	43[Link]	14[Link]	NA	Grade 2‐3 proteinuria (3), hypertension (3) grade 3 fatigue (2), epistaxis (2), joint pain (1), grade 2 pterygoid myositis (1), psychiatric symptoms (1)
Couec et al (2012)	7	3.1‐21.2 y	86[Link]	0	0	NA	Grade 1‐2 hypertension (4), proteinuria (1), lymphopenia (2), wound healing delay (2)[Link]
Packer et al (2009)	10	1 y 6 mo to 11 y 1 mo	50[Link]	20[Link]	20[Link]	NA	Transient leukoencephalopathy (1); grade 3 proteinuria (1)

BBT, bevacizumab‐based therapy; CNS, central nervous system; CR, complete response; MR, minor response; mo, months; NA, not available; PFS, progression‐free survival; PR, partial response; SD, stable disease; y, years old.

Clinical response.

Radiological response.

Data reported for 28 patients including high grade (HGG ‐ 12, LGG ‐ 7, neuroglial tumors ‐ 3, ependymoma ‐ 4, medulloblastoma ‐ 1, supratentorial PNET ‐ 1; no toxicity data were reported specifically for LGG patients).

## 2 PATIENTS AND METHODS

### 2.1 Study cohort and eligibility criteria

This study is a retrospective analysis of pediatric patients with refractory or progressive PLGG treated with bevacizumab‐based therapy (BBT) at two institutions (Royal Children's Hospital, Melbourne and Perth Children's Hospital, Perth) over the period of 2014 ‐ 2017. The aim was to analyse the objective response, progression‐free survival, and BBT‐related toxicities. Patients were identified using hospital databases and relevant clinical data, and treatment regimens were extracted from the patient's charts. The study was conducted with institutional ethics approval. Children aged 0‐18 years old at the time of diagnosis, with or without NF1 and with at least one documented progression, were eligible for the study. The patients were highly selected for given possible functional morbidity from progressive disease and often extensive pretreatment. Patients fulfilling clinical criteria for NF1 were formally genetically tested, and all available tissue samples were examined for BRAF‐MAPK pathway alterations. Patients without histological diagnosis were included when the clinical and radiological features were consistent with low‐grade glioma.

### 2.2 Treatment

Bevacizumab was added to the 1st to 6th line of therapy. Several chemotherapy regimens were used in our cohort include carboplatin/bevacizumab, vinblastine/bevacizumab, combination of vincristine/irinotecan/bevacizumab, combination of vincristine/carboplatin/bevacizumab, irinotecan/bevacizumab, lomustine (CCNU)/bevacizumab, and single agent bevacizumab. Bevacizumab was administered as a single IV dose of 10 mg/kg every 2 weeks, as previously described by Packer et al.^42^ Duration of treatment was defined in cycles, where one cycle was equal to completion of 2 doses of bevacizumab. Length of therapy was variable and depended on response and adverse event profile. Adverse events were assessed and graded according to Common Terminology Criteria for Adverse Events (CTCAE), version 4.03.^48^

### 2.3 Evaluation of response

An MRI brain with closest interval before commencement of bevacizumab was chosen as a baseline scan. Radiological response was assessed on MRIs at 3 months, end of treatment, and latest, if available. MRI scans were performed as per the institutional protocol, including 3‐plane (axial, sagittal and coronal) T1 precontrast, T1 postcontrast, FLAIR and T2‐weighted sequences. Largest bidirectional tumor area was measured on either axial, sagittal or coronal sequence. CR (complete response) was complete resolution of tumor, PR (partial response) was greater than or equal to 50% reduction in the largest bidirectional tumor area, MR (minor response) was 25%‐49% reduction in tumor area, SD (stable disease) was <25% reduction or <25% enlargement of largest bidirectional tumor area, and PD (progressive disease) was greater than or equal to 25% enlargement in bidirectional tumor measurement.^16^

Formal neurological and visual assessment information was collected from the visits closest in interval to the MRI timepoints for clinical response evaluation. Clinical or/and radiological response was used to determine the objective response.

### 2.4 Statistical analysis

Data was collected and presented as total number, percent, mean, and median as appropriate for the type of data. Survival analysis was done using Kaplan‐Meier method. Progression‐free survival (PFS) was defined as the time interval from BBT to the time of disease progression or recurrence, to the last follow‐up, or to death occurrence from any cause.

## 3 RESULTS

### 3.1 Cohort characteristics

In this study, we report 15 patients diagnosed with progressive PLGG who commenced BBT during the period between July of 2014 and June of 2017. Patient characteristics, therapies prior to commencement of BBT and BBT regimens are described in Table 2. Median age at initial diagnosis was 7 years old (range 3 months to 15 years old) and patients were treated with bevacizumab at median age of 11.1 years (range from 5.1 to 18 years of age). There were nine males and six females. In six patients (40%), tumors were in chiasmatic/hypothalamic region, four had midline tumors, two patients had disseminated disease, and one each had hemispheric, posterior fossa and spinal involvement. Histological diagnosis was available in 12/15 patients with following pathologies: pilocytic astrocytoma [n = 9], pleomorphic xanthoastrocytoma [n = 1], ganglioglioma [n = 1] and glioneuronal tumor [n = 1]. Three patients were not biopsied, and the diagnoses of low grade chiasmatic/hypothalamic tumor were made based on the clinical and radiological findings. Two of these patients had fulfilled clinical criteria for NF1 and had genetically confirmed diagnosis, and one patient did not satisfy NF1 clinical criteria and was not tested. In seven patients, sufficient tissue was available for BRAF‐MAPK pathway alterations testing: two had BRAF‐KIAA1549 fusion, two had BRAF V600E mutation, one patient was tested positive for RAF1 mutation (Noonan‐like syndrome) and two patients had no alterations. None of the patients with pilocytic astrocytoma had BRAF V600E mutations.

**Table 2:** Patient cohort characteristics, previous therapies, and current bevacizumab combinations

Patient ID	Gender	Age at Dx (y)	Age at BBT Start (y)	Tumor Location	Histology	RAF/MAPK Pathway	Previous Therapies	Bevacizumab Combinations
PLGG1	Male	4	5	Chiasmatic/Hypothalamic	Pilocytic astrocytoma	No data	Carbo/VCR (VBL)	Irinotecan/Bev
							Carbo/VCR	
PLGG2	Female	13	17	Chiasmatic/Hypothalamic	Pilocytic astrocytoma	Negative	Carbo	Irinotecan/VCR/Bev
							Carbo/VCR	
PLGG3	Male	7	16	Midline	Pilocytic astrocytoma	BRAF‐KIAA1549 fusion	Carbo	VBL/Bev
							TMZ	
							TPCV	
PLGG4	Male	<1	13	Chiasmatic/Hypothalamic	Not biopsied	NF1	Carbo VBL	Bev
PLGG5	Female	13	18	Hemispheric	Pleomorphic xanthoastrocytoma	BRAF V600E mut	Surgery	CCNU/Bev
							Surgery +Focal Rx	
							TPC(‐V)	
PLGG6	Male	7	18	Midline	Ganglioglioma	BRAF V600E mut	Carbo	Carbo/Bev
							TMZ	VBL/Bev
							Focal Rx	
							Irinotecan/Bev	
							Dabrafenib	
PLGG7	Female	11	12	Disseminated	Pilocytic astrocytoma	No data	Carbo/VCR	Carbo/VCR/Bev
PLGG8	Male	5	5	Midline	Pilocytic astrocytoma	BRAF‐KIAA1549 fusion	Carbo	Carbo/Bev
PLGG9	Female	3	6	Chiasmatic/Hypothalamic	Not biopsied	NF1	Carbo/VCR	VBL/Bev
							VBL	
PLGG10	Female	6	11	Spinal	Pilocytic astrocytoma	No data	Carbo	VBL/Bev
PLGG11	Male	15	15	Chiasmatic/Hypothalamic	Pilocytic astrocytoma	No data	Carbo	VBL/Bev
PLGG12	Male	9	11	Disseminated	Glioneuronal	RAF1mut (Noonan‐like syndrome)	Carbo	VBL/Bev
PLGG13	Female	4	9	Posterior fossa	Pilocytic astrocytoma	Negative	Carbo/VCR	Carbo/Bev
							VBL	
PLGG14	Male	1	10	Chiasmatic/Hypothalamic	Not biopsied	No data	Carbo/VCR	Carbo/Bev
							VBL	
							Focal Rx	
PLGG15	Male	9	11	Midline	Pilocytic astrocytoma	No data	Carbo	Carbo/Bev

Bev, bevacizumab; Carbo, carboplatin; Dx, diagnosis; Rx, radiation therapy; TMZ, temozolomide; TPCV, thioguanine, procarbazine, lomustine, vincristine; TPC(‐V), thioguanine, procarbazine, lomustine (without vincristine); VCR, vincristine; VBL, vinblastine; y, years.

Thirteen patients demonstrated both radiological and clinical progression, one patient had radiological progression and one patient only had isolated progressive visual deterioration with stable radiological disease prior to start of bevacizumab. Clinically, six patients had worsening of motor function, seven experienced progressive visual impairment and one being blind from the time of previous progression and four had worsening endocrinopathies (hypothalamic obesity with primary ovarian failure [n = 1], premature adrenarche [n = 1], panhypopituitarism [n = 1], syndrome of inappropriate antidiuretic hormone secretion (SIADH) with hypothalamic dysfunction [n = 1]).

### 3.2 Treatment

In 40% of the cases (six patients), bevacizumab was used as the 2nd line of therapy, in five cases bevacizumab was used as the 3rd line and in three cases as the 4th line of therapy, and in one patient, bevacizumab was used to treat the 6th progression. In one patient, bevacizumab was started upfront due to precipitous visual decline. Three patients had focal radiation as part of their treatment prior to bevacizumab.

Six patients were treated with the combination of vinblastine/bevacizumab, four patients received carboplatin/bevacizumab, and one each were treated with the combination of carboplatin/vincristine/bevacizumab, irinotecan/bevacizumab, irinotecan/vincristine/bevacizumab, and CCNU/bevacizumab at the discretion of the treating physician. One patient was treated with single agent bevacizumab. The conventional pediatric doses of other chemotherapeutic agents were used.

### 3.3 Radiological and clinical assessment

Patient details at commencement of BBT, clinical, and radiological responses and outcomes are described in Table 3. At the 3‐month assessment, 100% of patients demonstrated some degree of radiological response. No further worsening of clinical symptoms was observed, 4/8 patients had visual acuity improvement and 4/6 had improvement in motor function.

**Table 3:** Patients’ characteristics at commencement of bevacizumab‐based therapy, treatment, and outcomes

Patient ID	Reason to start BBT	Duration of BBT therapy (mo)	Clinical response at 3 mo	Clinical response at last follow‐up	Radiological response at 3 mo	Radiological response at last follow‐up	Period of stability of BBT (mo)	Progression
PLGG1	Radiological progression Clinical: hypothalamic‐pituitary axis dysfunction with SIADH; bilateral optic nerve atrophy	5	Improvement of endocrine function	Normal endocrine function	SD	SD	27	No
PLGG2	Radiological progression Clinical: visual acuity decline, hypothalamic obesity and primary ovarian failure	5	Stable endocrine function; improved visual acuity and fields	Stable endocrine function; improved visual acuity and fields	SD Reduced contrast enhancement	PD	10	Yes
PLGG3	Radiological progression Clinical: new right‐sided weakness	8	Improvement	Worsening	MR	PD	3	Yes
PLGG4	Radiological progression Clinical: visual acuity decline	11	Stable	Improvement	SD	SD	15	No
PLGG5	Radiological progression Clinical: new left‐sided weakness, hyperreflexia, left‐sided clonus	8	Stable	Improvement	SD Enhancement pattern necrosis vs. PD	PD	NA[Link]	Yes
PLGG6	Radiological progression Clinical: left‐sided weakness, right‐sided cranial nerve VII partial palsy and facial asymmetry, right‐sided tongue wasting, bilateral nystagmus	7	Stable	Worsening	SD Changes in contrast enhancement pattern	SD	7	No
PLGG7	Radiological progression Clinical: visual acuity decline and visual field deficits	7	Improvement in visual acuity	Improvement in visual acuity and fields	MR	MR	On therapy	No
PLGG8	Radiological progression Clinical: new left‐sided weakness	9	Improvement	Improvement	PR	SD	7	No
PLGG9	Radiological progression Clinical: premature adrenarche	12	Stable; No further puberty progression	Stable; No further puberty progression	SD Complete resolution of enhancing component	SD	On therapy	No
PLGG10	Radiological progression Clinical: increased left‐sided weakness, loss of proprioception, unable to walk independently, clonus, change in bowel habits	7	Improvement	Stable	PR	MR	On therapy	No
PLGG11	Clinical: visual acuity decline, visual field deficit, left‐sided proptosis	6	Improvement in visual acuity and fields	Improvement in visual acuity and fields	SD	MR	On therapy	No
PLGG12	Radiological progression Clinical: visual acuity decline	6	Stable	Stable	SD Enhancement pattern changes	SD	On therapy	No
PLGG13	Radiological progression Clinical: motor dysfunction	1.5[Link]	Stable	Stable	MR (intracranial)/SD (spinal)	Mixed SD (solid)/PD (cystic)	7	Yes (cystic component)
PLGG14	Radiological progression Clinical: visual acuity decline, panhypopituitarism	9	Stable	Stable	SD (solid)/MR (cystic)	Mixed SD (solid)/PD (cystic)	5	Yes (cystic component)
PLGG15	Radiological progression Clinical: left‐sided dense hemiparesis, visual acuity decline	11	Improvement both motor and visual acuity	Improvement both motor and visual acuity	PR	PR	On therapy	No

BBT, bevacizumab‐based therapy; MR, minor response, PD, progressive disease;PR, partial response; SD, stable disease

Patient progressed on bevacizumab therapy.

BBT was stopped for surgical reasons—ventriculoperitoneal shunt insertion.

**Figure 1:**
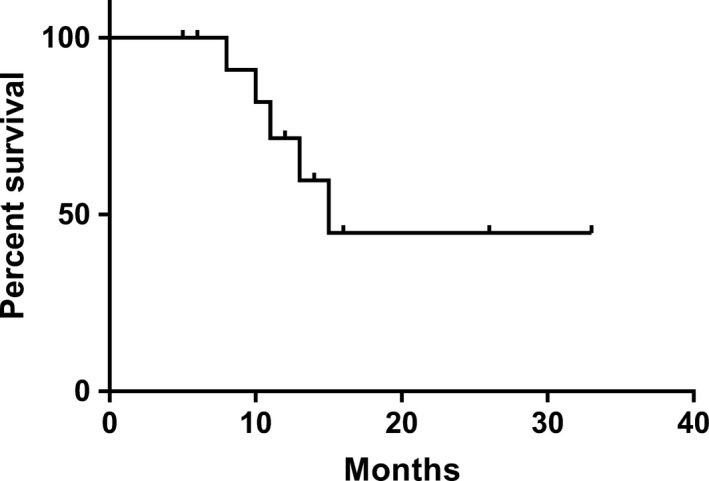
Progression‐free survival of the patients treated with bevacizumab‐based therapy (n = 15)

**Figure 2:**
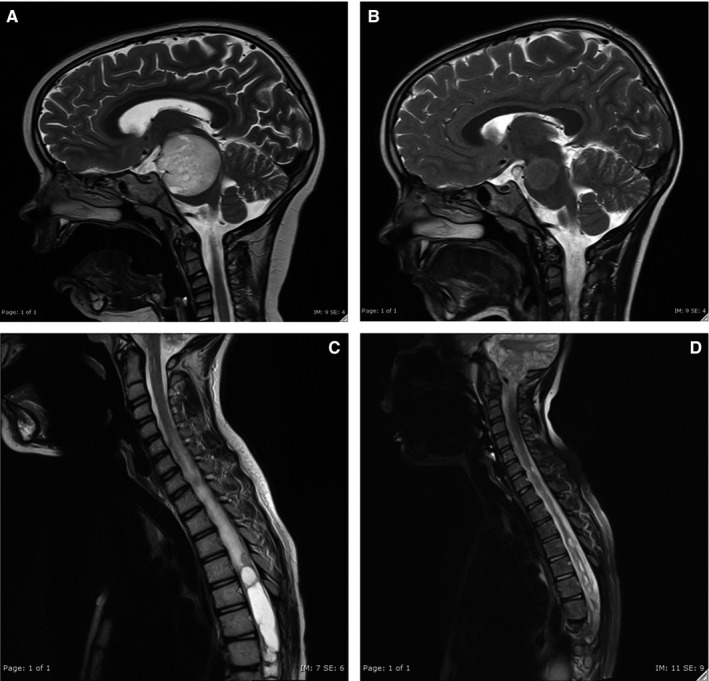
Radiological response to Bevacizumab therapy; MRI imaging at baseline PLGG (A) and PLGG10 (C) and 15 mo after completion of therapy PLGG8 (B) and after 6 mo of therapy PLGG10 (D)

Median follow‐up was 11 months (range 5‐33 months) from the start of the BBT. All patients remained alive at the time of the last follow‐up. PFS at 11 months was 71.5% ± 13.9%, however it dropped to 44.7% ± 17.6% at 15 months (Figure 1). At the last follow‐up, 40% (n = 6) of patients had radiologically stable disease, one patient had partial response and three had minor response (Figure 2). One patient had radiological progression at the end of therapy, two progressed at 3 and 10 months post completion of the therapy, respectively. Two patients showed mixed response with progression of cystic component and stable solid component at 4 and 6 months post cessation of treatment. No radiological complete response was documented in our cohort. Importantly, 6/8 patients had visual acuity improvement, 2/6 had both visual acuity improvement and partial recovery of hemianopia. Specifically, patient PLGG2 had LVA improved from 3/24 to 3/12 and RVA from hand motion (HM) to counting fingers (CF) at 50 cm with improvement of both visual fields, PLGG4 and PLGG7 both had visual recovery from HM to CF at 50‐80 cm which allowed for functional vision and significantly improved independent ambulation and quality of life in PLGG11. LVA was improved from 3/12 to 3/4.8 and improvement of visual field and decreased proptosis on the left were observed. Vision has remained stable without further deterioration in one patient [PLGG14] and was already lost during the previous progression [PLGG1] and no improvement was observed in the other. 2/4 had resolution of endocrine symptoms (normalization of the hypothalamic‐pituitary‐adrenal axis function [n = 2] with SIADH resolution [n = 1]) and 4/6 had motor function improvement. Patients PLGG3 and PLGG5 had worsening of motor function with radiologically progressive disease. Patient PLGG2 experienced improvement in visual acuity despite radiological progression. Patients PLGG13 and PLGG14 with isolated cystic progression remained clinically stable.

No relation between histology and BRAF status was observed in patients who experienced progression. Patients PLGG2 and PLGG13 had chiasmatic/hypothalamic pilocytic astrocytoma negative for BRAF alterations; PLGG3 had midline tumor with same histology, positive for BRAF‐KIAA1549; PLGG5 had PXA with BRAF V600E mutation which subsequently transformed to a high‐grade tumor months later and PLGG14 was not biopsied.

### 3.4 Length of therapy and adverse effects

The duration of therapy ranged from 1.5 to 12 months with the majority of patients (93%) completed five or more cycles of therapy. Five patients remained on therapy at the time of the last follow‐up. The limiting factors for continuation of the treatment were grade 4 proteinuria and hypertension in two patients, with preexisting hypertension required therapy in one of them and need for tumor‐related surgical intervention in two patients. Five out of 15 patients had grade 1‐2 proteinuria, 4/15 had mild neutropenia in the range of 1‐2.5 × 10^9^/L and one had grade 1 anemia—those patients continued therapy under close monitoring. No febrile neutropenic episodes were observed in our cohort. In patient PLGG14 with worsening of idiopathic hypertension and proteinuria, the therapy was stopped after 10 months. Resolution of both parameters occurred at 2 months mark post cessation of treatment. However, the tumor continued to progress posttreatment and he was restarted on carboplatin/bevacizumab regimen after 4 months break with support of double antihypertensive therapy. He remained stable clinically and his blood pressure was well controlled with grade 1‐2 proteinuria. Last MRI brain scan at 2 months post reinitiation of BBT demonstrated stable disease with decreased contrast enhancement. In patient PLGG15, treatment was stopped after 10 months due to grade 4 proteinuria and progressive worsening of hypertension despite being on two antihypertensive agents. Symptoms improved after 4 months of therapy, however patient remains on two antihypertensive agents with mild proteinuria. Tumor remains stable and no further therapy was initiated.

## 4 DISCUSSION

Bevacizumab‐base regimens have been increasingly utilized recently in both adult and pediatric glioma protocols with encouraging results.^38^, ^39^, ^40^, ^42^, ^43^, ^44^, ^45^ Low‐grade glioma in the pediatric population is a chronic recurrent disease known for multiple progressions and a heavy burden of morbidity, especially in individuals with unresectable tumors. It often requires multiple lines of therapy, including resorting to radiation treatment for tumor control.

In this selected cohort, we have demonstrated objective response with rapid clinical improvement in 10/15 (67%) patients who were previously treated with multiple therapeutic regimens including radiation therapy in three individuals. After 3 months of therapy, all the patients demonstrated a degree of radiological response. We also observed rapid recovery from debilitating clinical symptoms, including rapid improvement of vision in two patients and marked resolution of dense hemiparesis in another. Continuation of treatment demonstrated visual improvement in four additional patients and visual stabilization in the remaining two. Importantly, even though one of the patients with initial visual symptoms had radiological progression 10 months after cessation of therapy, her vision prebevacizumab therapy and remains stable at latest follow‐up, allowing this patient to retain functional vision. In our cohort, BBT demonstrated efficacy in patients with endocrinopathy secondary to hypothalamic/chiasmatic tumor location where we were able to achieve symptom resolution in two out of four patients and provide fast symptomatic improvement and stabilization in the remaining two. The difference in the response rates compared with other studies are likely related to the small sample size in each cohort and due to variation in the objective response evaluation where some studies used clinical response and others reported only radiological response (Table 1).

Of significance, we were able to successfully resume treatment in one of the patients after stopping therapy for 4 months due to toxicity. In this patient, treatment was limited by significant hypertension and proteinuria that resolved with cessation of bevacizumab. Following rapid tumor progression, with carefully chosen antihypertensives we were able to restart therapy leading to tumor stabilization and control of tumor‐related symptoms, suggesting that, although side effects of bevacizumab are not insignificant, they can be successfully managed to allow for continuation of therapy to avoid more damaging regimens. In addition, the efficacy of retreatment with bevacizumab did not appear to be decreased by previous exposure.^42^, ^45^

It was previously described in a small number of cases that monotherapy with bevacizumab has its own benefits, as removal of irinotecan from the treatment regimen decreases combined toxicity without significant effect on good outcomes.^43^ In our cohort, we have examined the role of bevacizumab in conjunction with the most frequently used PLGG therapeutic regimens, namely monotherapy with vinblastine or carboplatin or with a combination of carboplatin and vincristine. Eleven out of fifteen patients (73%) have received bevacizumab at the start of the regimen and then continued with the conventional schedule. Nine out of eleven patients (82%) had response to these combinations, which is comparable with previous reports of irinotecan/bevacizumab protocol.^42^, ^43^, ^44^ We did not observe an increase in toxicities beyond expected, with only mild neutropenia, likely related to vinblastine. The addition of bevacizumab to conventional carboplatin and vinblastine regiments is advantageous in providing rapid symptomatic control and sparing from devastating neurological deficits associated with radiotherapy, if acute clinical need arises. In general, the observed toxicities, including grade 4 hypertension and proteinuria, were reversible with cessation of the treatment. We did not encounter the severe hemorrhagic or thrombotic events reported in the adult population.^38^, ^39^, ^40^

All five patients with progression had the event <12 months post completion of bevacizumab, suggesting, in agreement with previous reports,^42^, ^43^, ^44^ that although therapy provides unarguable clinical benefits, the outcomes are not durable. No correlation of progression with any of the variables, including the presence or absence of PLGG associated molecular tumor markers, was established, with the exclusion of the case PLGG5 with histological diagnosis of BRAF V600E mutant pleomorphic xanthoastrocytoma. This patient had initial stabilization of her disease at three‐months assessment, and subsequent tumor progression after 8 months of therapy. At this time, the tumor was found to have undergone transformation to high‐grade glioma, which is in concordance with previous literature reports.^49^, ^50^ Both NF1 patients had stable disease and improvement of clinical symptoms with complete resolution of contrast enhancement in the previously enhancing portion of the lesion in PLGG9.

In conclusion, BBT in PLGG appears to be clinically beneficial with minimal risk of toxicity. Based on this success, our groups have adopted the use of BBT. We recommend its use as an adjunct to conventional chemotherapy where rapid tumor control is needed in cases of pending morbidity, in particular, loss of vision and spinal cord function in both the relapse and upfront settings. The optimal duration of therapy is not defined; however, it seems plausible to consider longer courses of therapy if tolerated. Bevacizumab can be effectively and safely combined with a number of regimens to allow flexibility of the patient treatment and re‐treatment. Because it appears that bevacizumab exerts the most noticeable effect on visual symptoms, prevalent in not routinely biopsied hypothalamic‐chiasmatic lesions irrespective of BRAF/MAPK pathway changes, it can be successfully used in OPG cases where BRAF‐ and MEK inhibitors are not available due to the unknown status of the pathway. Larger prospective studies are required to determine whether the response is conditional upon different histological and molecular characteristics of PLGG.

## CONFLICT OF INTEREST

None declared.
